# Supplementary material for: WNK1-dependent water influx is required for CD4+ T cell activation and T cell-dependent antibody responses
Source: Nat Commun. 2025 Feb 21;16:1857. doi: 10.1038/s41467-025-56778-x (PMC11845700; doi:10.1038/s41467-025-56778-x)
Supplement: Supplementary file 1 — Supplementary Information [file 41467_2025_56778_MOESM1_ESM.pdf]

Supplementary Information

Water influx is required for $CD4^+$ T cell activation and T cell-dependent antibody responses

Joshua Biggs O'May[1,3], Lesley Vanes[1], Leonard L. de Boer[1,2,4], David A. Lewis[1], Harald Hartweger[1,5], Simone Kunzelmann[1], Darryl Hayward[1,6], Miriam Llorian[1], Robert Köchl[1,7], Victor L. J. Tybulewicz[1]*

[1]The Francis Crick Institute, London, NW1 1AT, UK
[2]Imperial College, London W12 0NN, UK

*Correspondence to:

Victor L. J. Tybulewicz
The Francis Crick Institute,
1 Midland Road,
London, NW1 1AT, UK

Tel: +44 20 3796 1612
Email: Victor.T@crick.ac.uk

Present addresses:

[3] joshua.biggsomay@gmail.com
[4] Science for Life Laboratory, Department of Women's and Children's Health, Karolinska Institute, Box 1031, SE-171 21 Solna, Sweden
[5] Laboratory of Molecular Immunology, The Rockefeller University, 10065 New York, NY, USA
[6] GSK, Stevenage, SG1 2NY, UK
[7] Kings College London, London, SE1 9RT, UK

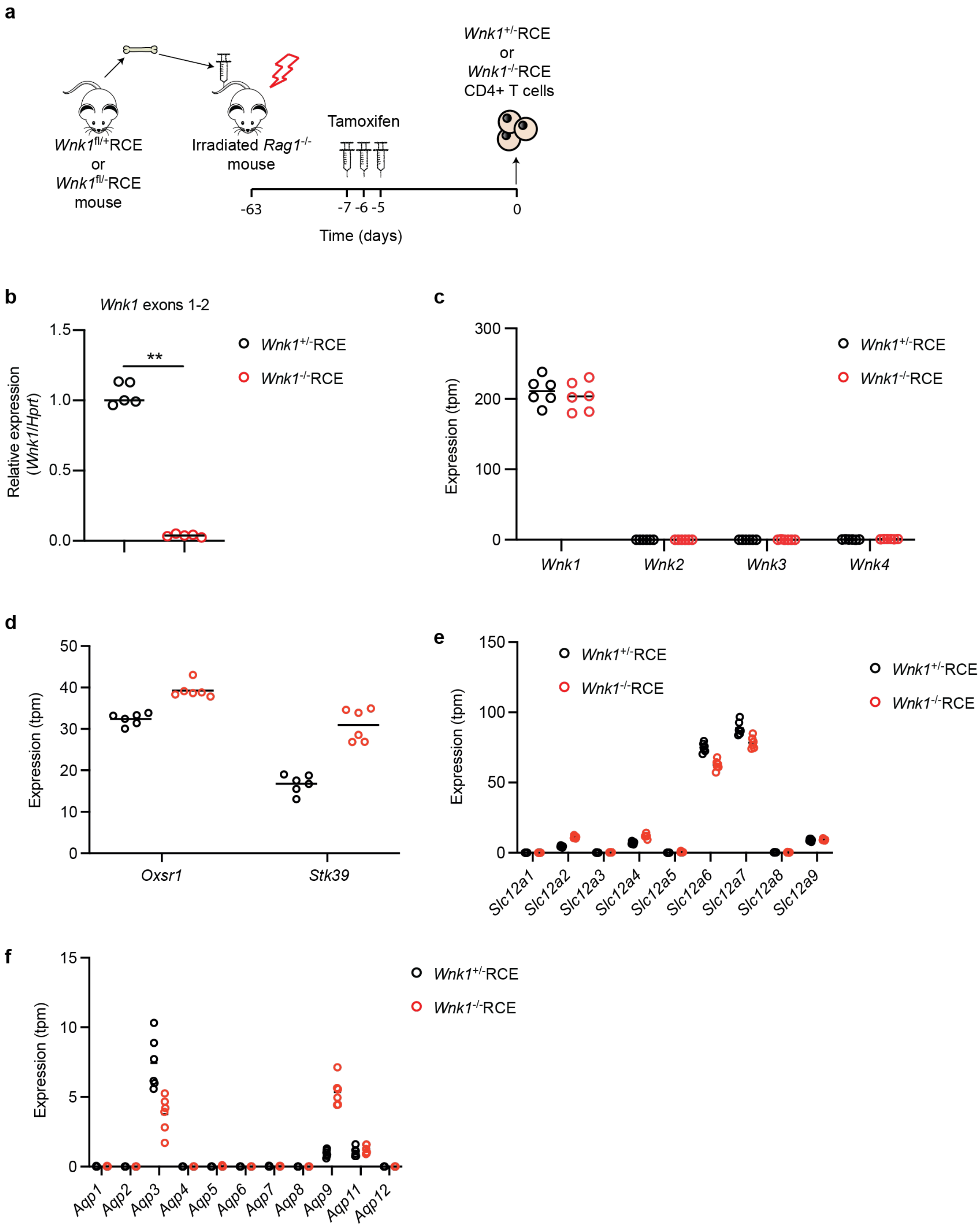

Supplementary Figure 1. Generation of WNK1-deficient T cells.

(**a**) Bone marrow from *Wnk1*$^{fl/+}$/RCE and *Wnk1*$^{fl/-}$/RCE mice was transferred into irradiated RAG1-deficient mice. At least 8 weeks later, the chimeras were treated with tamoxifen on three successive days. Lymph nodes were harvested 7 d after start of tamoxifen treatment

and WNK1-expressing (*Wnk1*$^{+/-}$RCE) or WNK1-deficient (*Wnk1*$^{-/-}$RCE) naive CD4^{+} T cells purified. (**b**) Graph of qPCR measurement of mRNA levels of *Wnk1* (exons 1-2) relative to *Hprt* in *Wnk1*$^{+/-}$RCE and *Wnk1*$^{-/-}$RCE naïve CD4^{+} T cells generated as in a, normalized to median level in *Wnk1*$^{+/-}$RCE T cells which was set to 1 (n = 5). (**c**) mRNA levels of *Wnk* genes in *Wnk1*$^{+/-}$RCE and *Wnk1*$^{-/-}$RCE CD4^{+} T cells determined by RNAseq (n=6) (Supplementary Data 1). Note that while exon 2 of *Wnk1* is efficiently deleted in *Wnk1*$^{-/-}$RCE CD4^{+} T cells, as shown in b, the remaining 27 exons are intact and still expressed, which is why the total amount of *Wnk1* mRNA is not decreased in the *Wnk1*$^{-/-}$RCE cells. The deletion of exon 2 from the *Wnk1* transcript removes part of the kinase domain and results in a frameshift, so the mutant allele destroys kinase activity and is most likely a null. (**d-f**) mRNA levels of *Oxsr1*, and *Stk39* genes (d), *Slc12a*-family genes (e) and *Aqp* genes (f) in *Wnk1*$^{+/-}$RCE and *Wnk1*$^{-/-}$RCE CD4^{+} T cells determined by RNAseq (n=6) (Supplementary Data 1). Note that the expression of *Slc12a*-family and *Aqp*-family genes in *Wnk1*$^{+/-}$RCE T cells is also shown in Figures 5d and 6d. tpm, transcripts per million reads. Each point represents a different mouse; horizontal lines show median; data pooled from 1 (c-f) or 2 (b) independent experiments. Statistical analysis carried out using the Mann-Whitney U test. ** $0.001 < p < 0.01$. Source data are provided as a Source Data file.

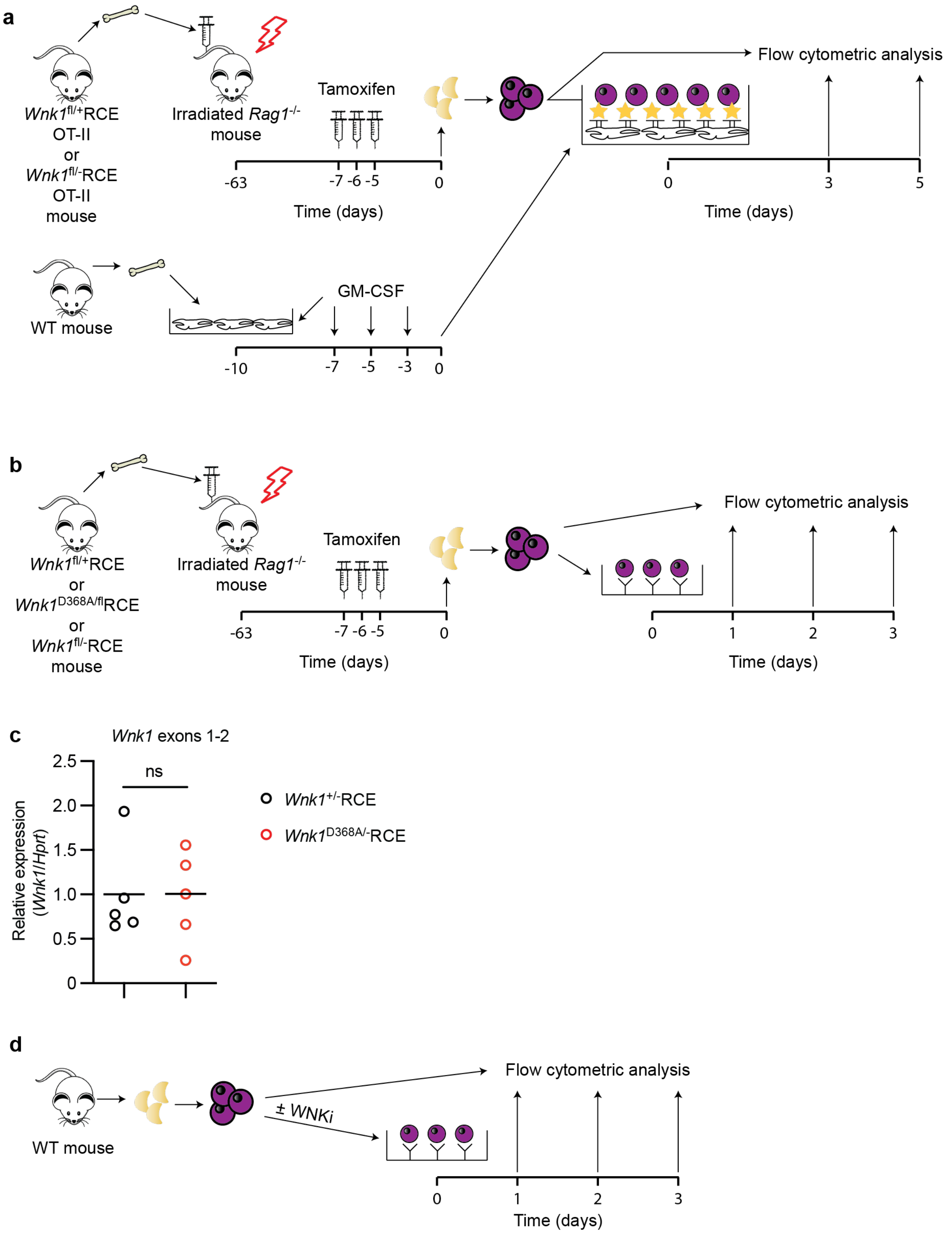

Supplementary Figure 2. *In vitro* analysis of activation of WNK1-deficient CD4^{+} T cells.

(**a**) Bone marrow from *Wnk1*$^{fl/+}$RCE OT-II and *Wnk1*$^{fl/-}$RCE OT-II mice was transferred into irradiated RAG1-deficient mice. At least 8 weeks later, the chimeras were treated with tamoxifen on three successive days to delete the floxed exon 2 of *Wnk1*, lymph nodes harvested 7 d after start of tamoxifen treatment and WNK1-expressing (*Wnk1*$^{+/-}$RCE OT-II) or WNK1-deficient (*Wnk1*$^{-/-}$RCE OT-II) naive CD4$^+$ T cells purified. APCs were generated from WT bone marrow cells cultured in GM-CSF containing medium for 10 d. T cells were labelled with CTV and co-cultured with APCs for up to 5 days, in the presence of indicated concentrations of $OVA_{323-339}$ peptide and analyzed by flow cytometry. (**b**) Bone marrow from *Wnk1*$^{fl/+}$RCE, *Wnk1*$^{fl/-}$RCE or *Wnk1*$^{D368A/fl}$RCE mice was transferred into irradiated RAG1-deficient mice. At least 8 weeks later, the chimeras were treated with tamoxifen on three successive days, lymph nodes harvested 7 d after start of tamoxifen treatment and WNK1-expressing (*Wnk1*$^{+/-}$RCE), WNK1-deficient (*Wnk1*$^{-/-}$RCE) or WNK1-D368A expressing (*Wnk1*$^{D368A/-}$RCE) naive CD4$^+$ T cells were purified. T cells were labelled with CTV and activated using plate-bound anti-CD3ε and anti-CD28 antibodies and analyzed by flow cytometry up to 3 d later. (**c**) Graph of qPCR measurement of mRNA levels of *Wnk1* (exons 1-2) relative to *Hprt* in *Wnk1*$^{+/-}$RCE and *Wnk1*$^{D368A/-}$RCE naïve CD4$^+$ T cells generated as in Supplementary Figure 2b, normalized to median level in *Wnk1*$^{+/-}$RCE T cells which was set to 1 (n = 5). Each point represents a different mouse; horizontal lines show median; data pooled from 2 independent experiments (c). Statistical analysis carried out using the Mann-Whitney U test; ns, not significant. (**d**) WT naïve CD4$^+$ T cells were labelled with CTV, activated on anti-CD3ε and anti-CD28 antibodies in the presence or absence of WNKi and analyzed at indicated time points. WNKi, WNK inhibitor (WNK463). Source data are provided as a Source Data file.

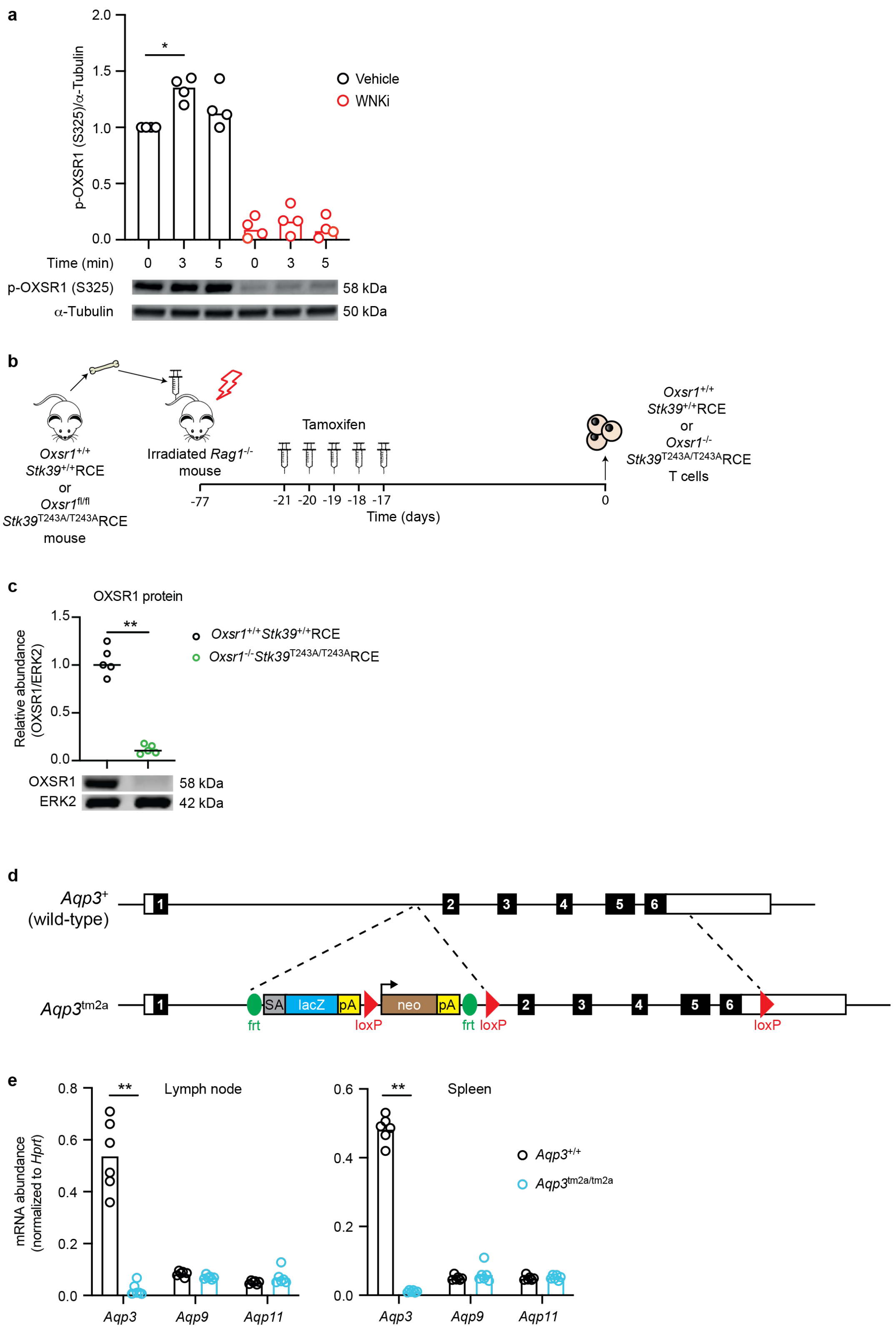

Supplementary Figure 3. Generation of CD4^{+} T cells with mutations in both *Oxsr1* and *Stk39*.

(**a**) Graph and example immunoblot of p-OXSR1 (S325) and α-tubulin from lysates of naïve CD4$^+$ T cells stimulated by cross-linking soluble anti-CD3ε and anti-CD28 antibodies for indicated timepoints (n=4). Data normalised to vehicle-treated sample at time 0 min which was set to 1. WNKi, WNK inhibitor (WNK463). Protein sizes in kDa. (**b**) Bone marrow from *Oxsr1*$^{+/+}$*Stk39*$^{+/+}$RCE and *Oxsr1*$^{fl/fl}$*Stk39*$^{T243A/T243A}$RCE mice was transferred into irradiated RAG1-deficient mice. At least 8 weeks later, the chimeras were treated with tamoxifen on five successive days to delete the floxed exons 9 and 10 of *Oxsr1*, lymph nodes harvested 21 d after start of tamoxifen treatment and *Oxsr1*$^{+/+}$*Stk39*$^{+/+}$RCE or *Oxsr1*$^{-/-}$*Stk39*$^{T243A/T243A}$RCE naive CD4$^+$ T cells purified. (**c**) Abundance of OXSR1 in *Oxsr1*$^{+/+}$*Stk39*$^{+/+}$RCE or *Oxsr1*$^{-/-}$*Stk39*$^{T243A/T243A}$RCE CD4$^+$ T cells was determined by immunoblotting total cell lysates for OXSR1 and ERK2 and normalizing the ratio of OXSR1 to ERK2 to the median value in *Oxsr1*$^{+/+}$*Stk39*$^{+/+}$RCE T cells which was set to 1 (example immunoblot shown below graph) (n = 5). (**d**) Diagram showing the structure of the wild-type (*Aqp3*$^+$) and *Aqp3*$^{tm2a(EUCOMM)Wtsi}$ (*Aqp3*^{tm2a}) *Aqp3* alleles. The latter contains a splice acceptor (SA), lacZ gene, polyadenylation site (pA), a neo gene with its own promoter, and another pA site inserted into intron 1. Rectangles depict exons 1-6 with coding and non-coding regions in black and white respectively. Also indicated are integrated frt and loxP sites. (**e**) Graph of qPCR measurement of mRNA levels of *Aqp3*, *Aqp9* and *Aqp11* in lymph node or splenic CD4$^+$ T cells of the indicated genotypes, normalized to expression of *Hprt* (n=6). Each point represents a different mouse; horizontal lines or columns indicate median; data shown from 1 experiment representative of 2 independent experiments (c) or pooled from 3 (a) or 2 (e) independent experiments. Statistical analysis carried out using the Mann-Whitney U test; * $p<0.05$, ** $0.001 < p < 0.01$. Source data are provided as a Source Data file.

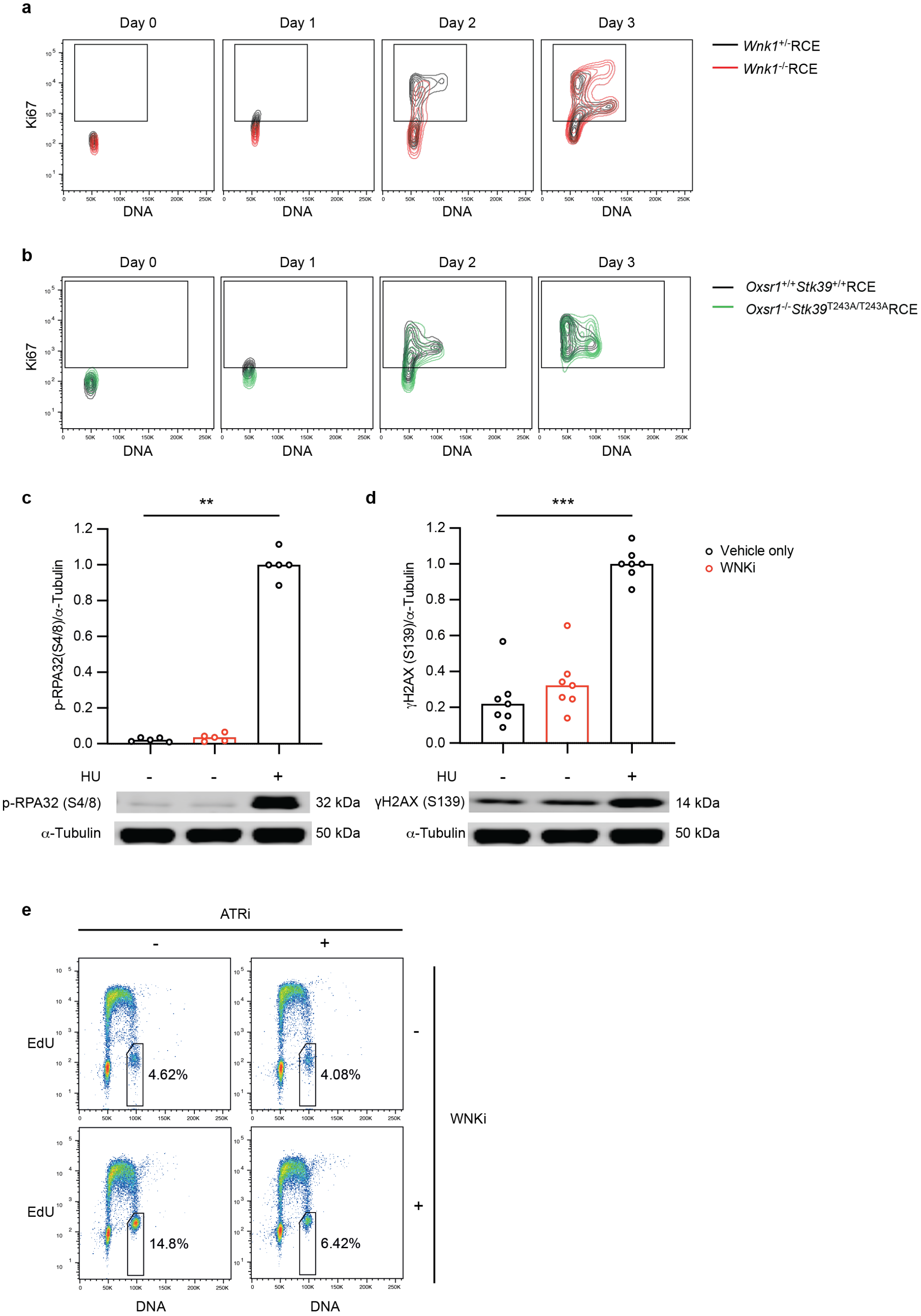

Supplementary Figure 4. Delayed TCR/CD28-induced entry into G1 in CD4⁺ T cells with mutations in *Wnk1*, *Oxsr1* and *Stk39*.

(**a, b**) Example flow cytometric density plots of Ki67 expression and DNA content of CD4$^+$ T cells of the indicated genotypes activated on plate-bound anti-CD3ε and anti-CD28 antibodies for the indicated times. Gates show Ki67$^+$ populations used to generate data in Figures 3a, f. (**c**, **d**) Graphs showing abundance of p-RPA32 (S4/S8) (c) and γH2AX (S139) (d) relative to Tubulin in CD4$^+$ T cells activated on plate-bound anti-CD3ε and anti-CD28 antibodies for 3 d in the presence of WNKi or vehicle only (c, n=5; d, n=7). Where indicated, 4 mM hydroxyurea (HU) was added for the final 2 h of culture. Amounts of p-RPA32 and γH2AX were determined by immunoblotting total cell lysates with antibodies to these phosphorylated proteins and to Tubulin and normalizing the resulting pRPA32/tubulin and γH2AX/tubulin ratios to the median ratio in HU-treated cells which was set to 1. Note the same tubulin loading control is used in both c and d. Columns show median; each point represents a single mouse. Protein sizes in kDa. (**e**) Representative flow cytometric pseudocolor plots of DNA and EdU content of single, live CD4$^+$ T cells activated for 72 h on anti-CD3ε- and anti-CD28-coated cell culture dishes. Cells were treated with WNKi for 72 h or ATRi for 6 h where indicated and pulsed with EdU for the final 30 min of the culture. Gates indicate cells in G2/M used to generate data in Figure 4j, numbers indicate percentage of cells falling into the gate. Data pooled from 3 independent experiments (c, d). Statistical analysis carried out using Mann-Whitney U test. ** $0.001 < p < 0.01$; ***$0.0001<p<0.001$. Source data are provided as a Source Data file.

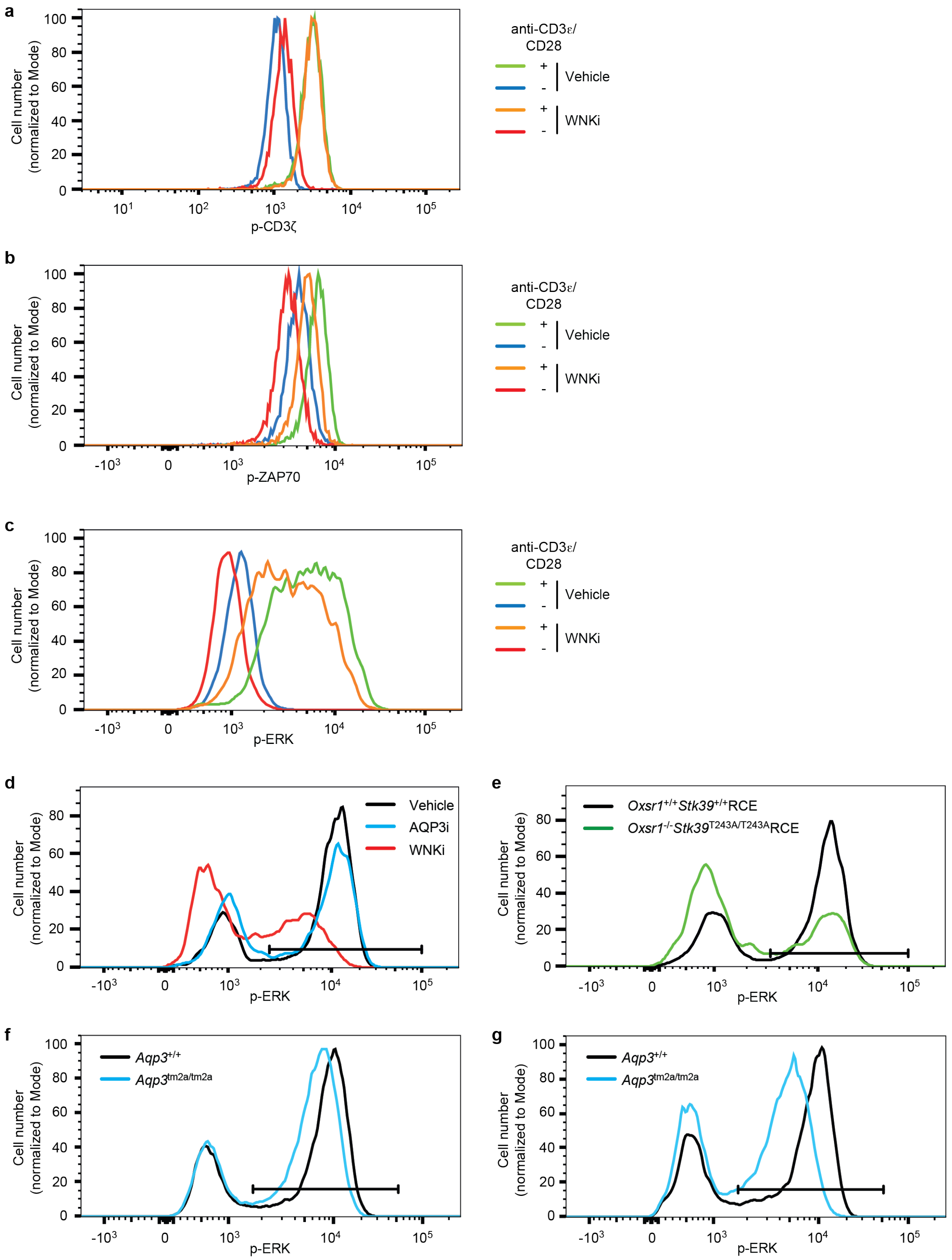

Supplementary Figure 5. Flow cytometric analysis of early TCR signaling.

(**a-g**) Example flow cytometric histograms of intracellular staining with antibodies for p-CD3ζ (Y142) (a), p-ZAP70 (Y319) (b), and p-ERK1/2 (T202/Y204) (c-g) of $CD4^+$ T cells from lymph nodes (a-f) or spleen (g) that had been activated or not with soluble anti-CD3ε and anti-CD28 antibodies for 2 min (a-c) or with plate-bound antibodies for 1 h (d-g). Cells were treated with inhibitors as indicated. Cells were from C57BL/6J mice (a-d) or were of the indicated genotypes (e-g). Gating for p-ERK high cells is shown (d-g). WNKi, WNK inhibitor (WNK463); AQP3i, AQP3 inhibitor (DFP00173).

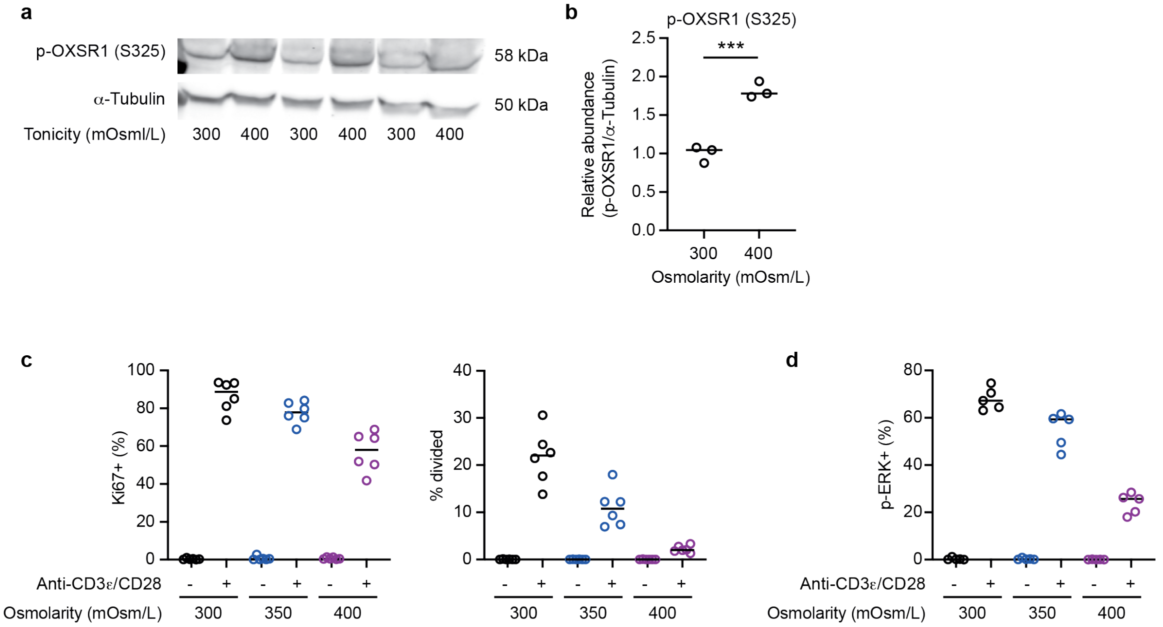

Supplementary Figure 6. Effect of hypertonicity on T cell activation.

(**a, b**) Immunoblot of cell lysates from CD4$^+$ T cells treated with isotonic (300 mOsm/L) or hypertonic (400 mOsm/L) for 5 min and probed with antibodies to p-OXSR1 (S325) and α-tubulin (a). Protein sizes in kDa. Graph showing abundance of p-OXSR1 (S325) normalized to α-tubulin and then to the signal in cells in isotonic medium in cells at two different osmolarities quantified from the immunoblot in a (n=3) (b). (**c, d**) CD4$^+$ T cells activated with plate-bound anti-CD3ε and anti-CD28 antibodies in isotonic (300 mOsm/L) or hypertonic (350 or 400 mOsm/L) medium for 2 days (c) or 1 h (d). Graphs show % Ki67+ cells (n=6), % of cells that have divided (n=6) and % p-ERK$^+$ cells (n=5). Each dot is an independent biological replicate, lines indicate median. Data is from a single experiment (b) or pooled from 2 experiments (c, d). Statistical analysis carried out using the Mann-Whitney U test; *** p<0.001. Source data are provided as a Source Data file.

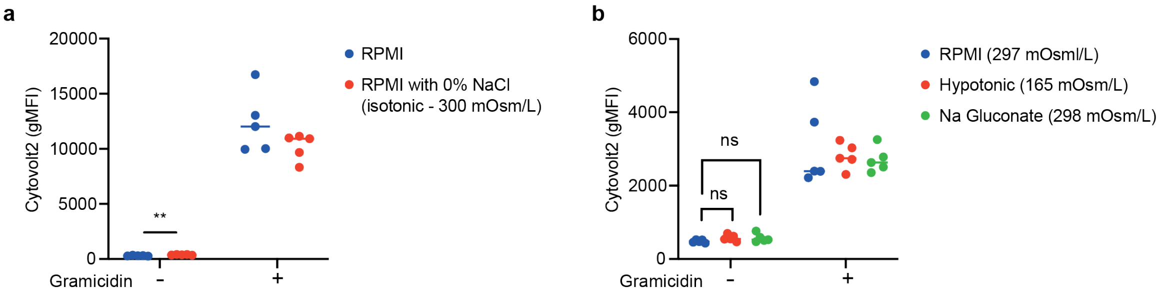

Supplementary Figure 7. Altered NaCl concentrations and hypotonicity do not affect membrane potential.

(**a, b**) Graphs showing geometric MFI (gMFI) of Cytovolt2 dye fluorescence, a measure of membrane potential, in CD4^{+} T cells in RPMI or isotonic RPMI with 0% NaCl used in Figure 5b (a) or in cells in RPMI, or in isotonic RPMI in which NaCl had been replaced with Na gluconate or hypotonic medium as used in Figures 5c and 7a, b respectively (b) (n=5). As a positive control, cells were treated with gramicidin to depolarize the membrane. Data is pooled from 2 experiments. Statistical analysis carried out using the Mann-Whitney U test; ** $0.001 < p < 0.01$; ns, not significant. Source data are provided as a Source Data file.

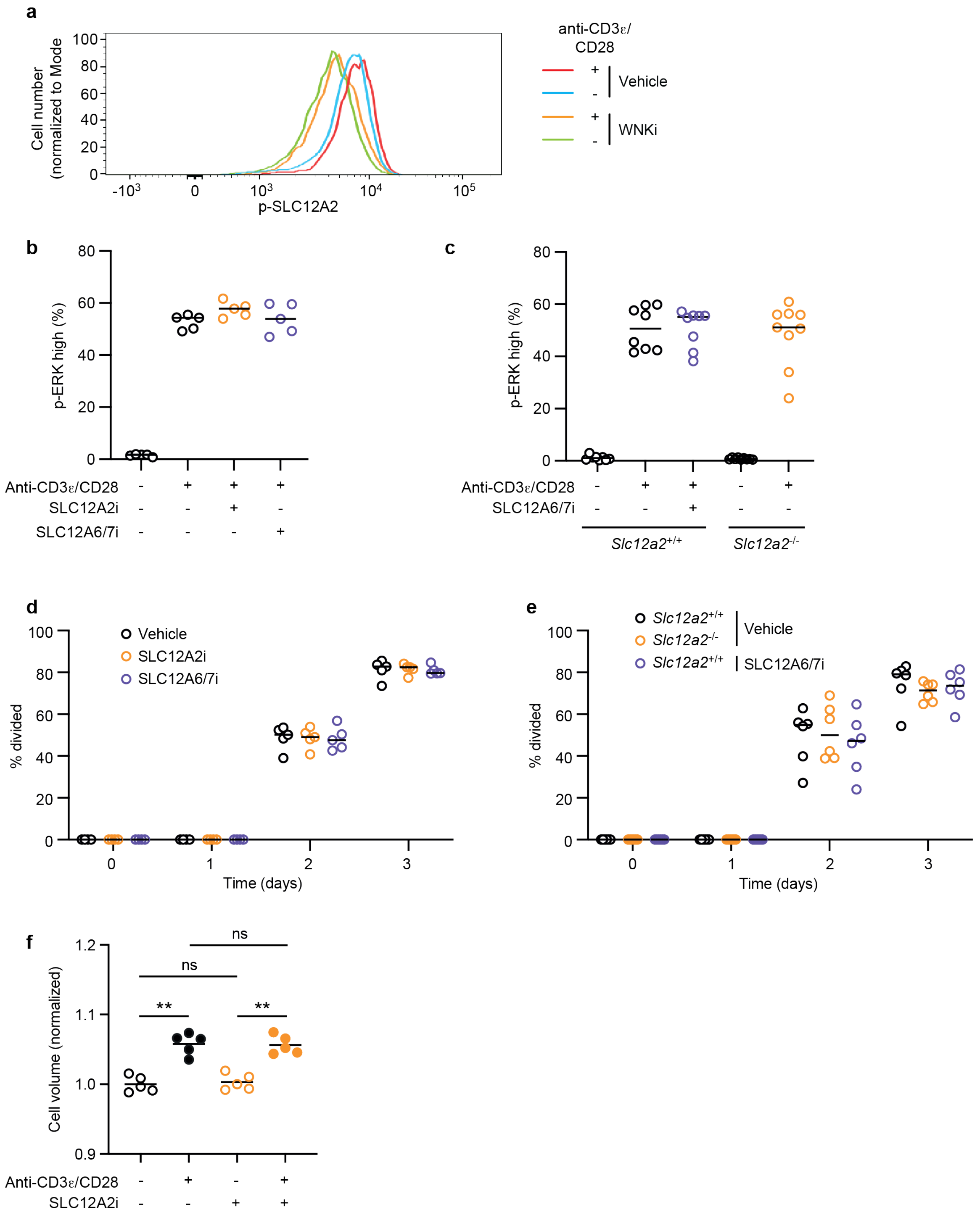

Supplementary Figure 8. SLC12A2, SLC12A6 and SLC12A7 are not required for TCR-induced activation or proliferation.

(**a**) Example flow cytometric histograms of intracellular staining with an antibody for p-SLC12A2 (T203/T207/T212) in CD4$^+$ T cells that had been activated or not with plate-

bound antibodies for 1 h in the presence or absence of WNKi. (**b-c**) Percentage of p-ERK high CD4$^+$ T cells that had been activated or not with plate-bound antibodies for 1 h in the presence or absence of inhibitors. Cells were from C57BL/6J mice (b) or of the indicated genotypes (c) (b, n=5; c, *Slc12a2*$^{+/+}$ n=8, *Slc12a2*$^{-/-}$ n=9). (**d-e**) CTV-labelled CD4$^+$ T cells were incubated with plate-bound antibodies for the indicated times and CTV dilution was used to determine the percentage of cells that had divided at least once (d, days 0, 1: n=4, days 2, 3: n=5; e, n=6). (**f**) Volume of naïve WT CD4$^+$ T cells activated for 1 h in the presence of vehicle only or SLC12A2i on plate-bound anti-CD3ε and anti-CD28 antibodies where indicated, determined using a Casy® cell counter and normalized to the mean volume of cells in the absence of stimulation or inhibitor. Each dot represents the modal volume of ≥200 cells (n=5). WNKi, WNK inhibitor (WNK463); SLC12A2i, SLC12A2 inhibitor (bumetanide); SLC12A6/7i, SLC12A6 and SLC12A7 inhibitor (DIOA). Dots represent cells from a single mouse, lines indicate median (b-e) or mean (f). Data pooled from 2 (b-e) or 1 (f) independent experiments. Statistical analysis with a Mann-Whitney test showed that SLC12A2i and SLC12A6/7i and the genetic loss of *Slc12a2* had no significant effect on the percentage of p-ERK high cells or the percentage of cells that had divided. ** $0.001 < p < 0.01$; ns, not significant. Source data are provided as a Source Data file.

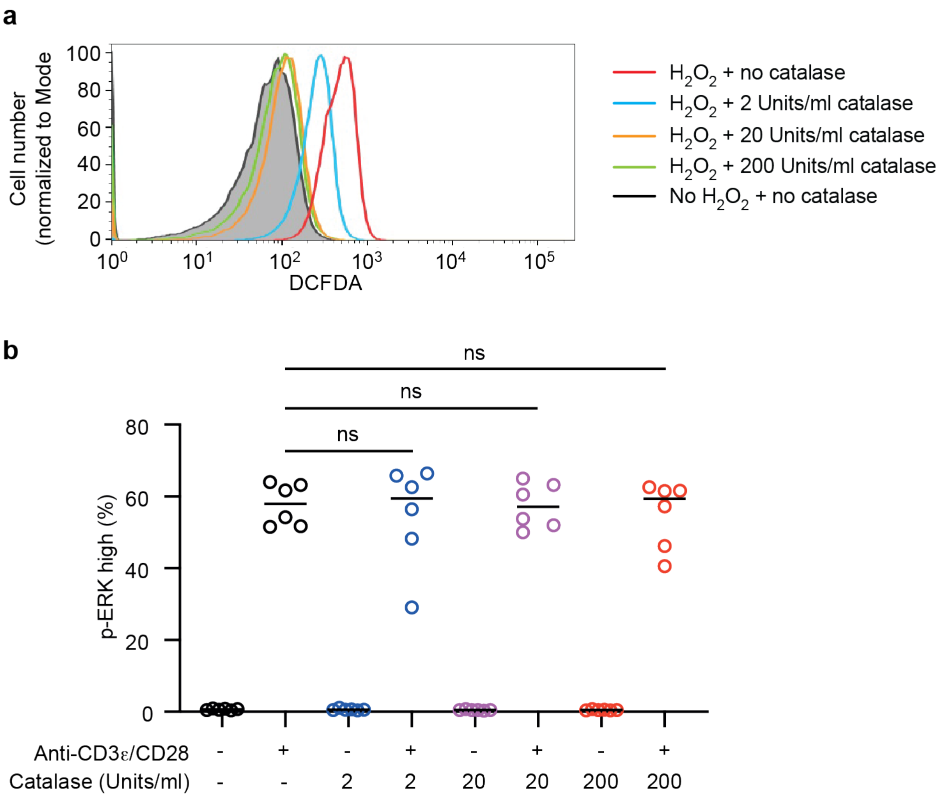

Supplementary Figure 9. Extracellular H_2O_2 is not required for TCR-induced activation.

(**a**) Representative flow cytometry plot of mouse naïve $CD4^+$ T cells stained with the reactive oxygen species indicator DCFDA, pre-incubated with catalase at the indicated concentrations. Where indicated, cells were treated with 1 mM H_2O_2 for 30 min and analyzed using flow cytometry. (**b**) $CD4^+$ T cells were activated on plate-bound anti-CD3ε and anti-CD28 antibodies for 1 h, as indicated, in the presence of different concentrations of catalase. Graph shows percentage p-ERK high cells (n=6). Data pooled from 2 experiments (b). Statistical analysis with a Mann-Whitney test showed that catalase had no significant effect (ns) on the percentage of p-ERK high cells. Source data are provided as a Source Data file.

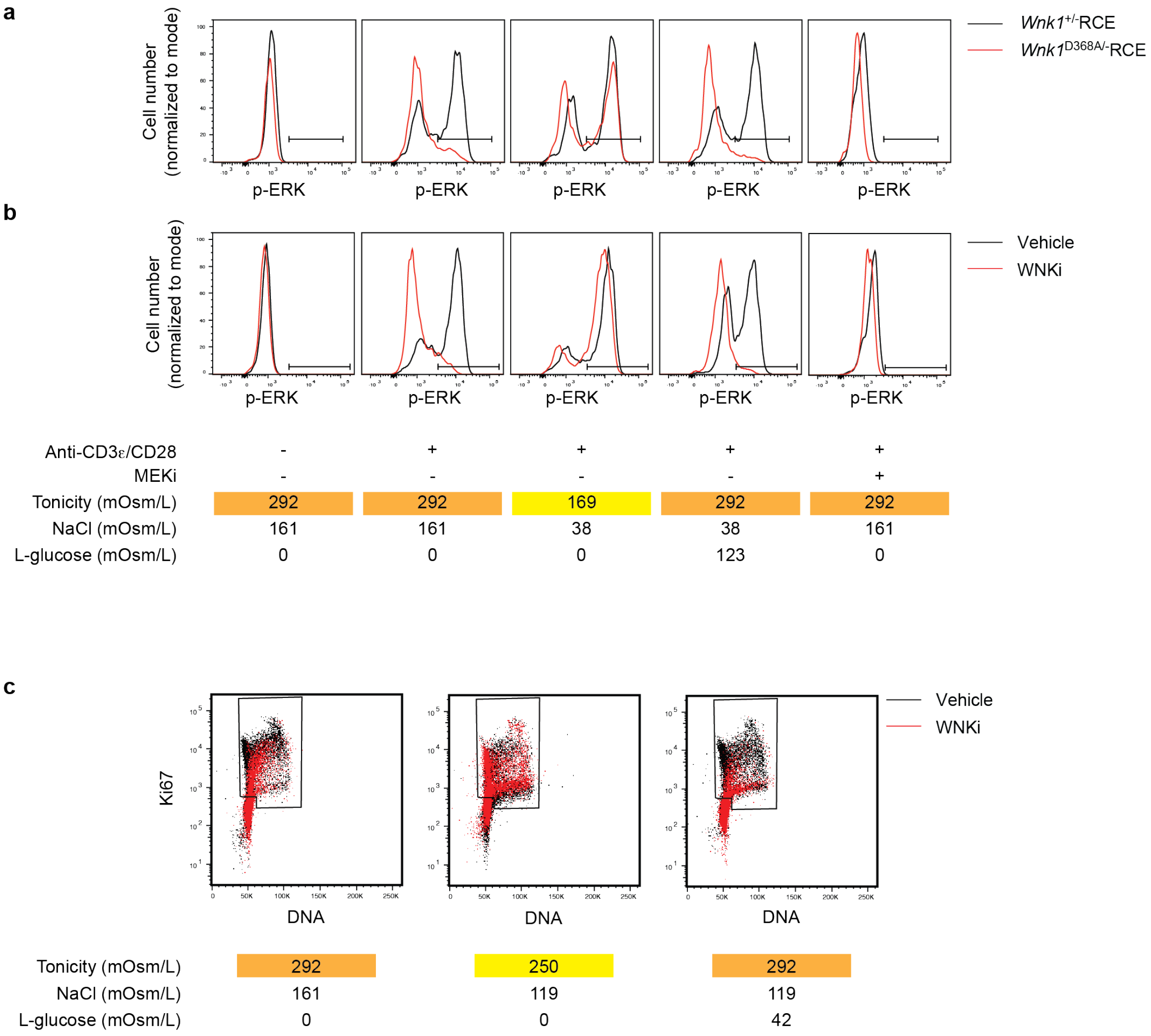

Supplementary Figure 10. WNK1-dependent water influx is required for TCR signaling and G1 entry.

(**a**, **b**) Histograms of phospho-ERK1/2 (p-ERK, T202/Y204) abundance in CD4$^+$ T cells activated for 1 h on plates coated with anti-CD3ε and anti-CD28 antibodies at the tonicities indicated and in the presence of MEKi or vehicle only, measured by flow cytometry. Histograms show a comparison of *Wnk1*$^{+/-}$RCE and *Wnk1*$^{D368A/-}$RCE CD4$^+$ T cells (a) or WT CD4$^+$ T cells treated with WNKi or vehicle (b). Gate indicates p-ERK high cells. (**c**) Example dot plots of Ki67 expression and DNA content of WT CD4$^+$ T cells activated for 2 d on anti-CD3ε and anti-CD28 antibodies in the presence of WNKi or vehicle only at the

tonicities indicated, made by altering NaCl and L-glucose concentrations, measured by flow cytometry; gate indicates Ki67^{+} cells, excluding Ki67^{-} cells in G0. WNKi, WNK inhibitor (WNK463).

Supplementary Table 1. Antibodies used in this study.

**Antigen**	**Clone**	**Supplier**	**Dilution**
ERK	3A7	Cell Signaling Technology	1:1000 (Immunoblot)
OXSR1	Polyclonal	MRC PPU Reagents and services	1:1000 (Immunoblot)
p-CHK1 (S345)	133D3	Cell Signaling Technology	1:1000 (Immunoblot)
p-OXSR1 (S325)	Polyclonal	MRC PPU Reagents and Services	1:1000 (Immunoblot)
p-RPA32 (S4/8)	Polyclonal (#A300-245A)	Bethyl Laboratories	1:1000 (Immunoblot)
α-Tubulin	TAT-1	Cell Services STP, Crick	1:15000 (Immunoblot)
γH2AX (S139)	JBW301	Merck Millipore	1:10000 (Immunoblot)
Mouse IgG	IRDye® 800CW	LI-COR Biosciences	1:4000 (Immunoblot)
Mouse IgG	Alexa Fluor 680	ThermoFisher Scientific	1:4000 (Immunoblot)
Rabbit IgG	IRDye® 800CW	LI-COR Biosciences	1:4000 (Immunoblot)
Rabbit IgG	Alexa Fluor 680	ThermoFisher	1:4000

		Scientific	(Immunoblot)
Sheep IgG	Alexa Fluor 680	ThermoFisher Scientific	1:4000 (Immunoblot)
CD3ε	145-2C11	Tonbo Biosciences	1:125 (plate-bound stimulation)<br>1:50 (soluble stimulation)
CD28	37.51	Biolegend	1:250 (plate-bound stimulation)<br>1:100 (soluble stimulation)
Hamster IgG (H&L chain)	Polyclonal (#ABIN101378)	Antibodies-online.com	1:25 (soluble stimulation)
CD4	GK1.5	ThermoFisher Scientific	1:200 (FACS)
CD45.1	A20	ThermoFisher Scientific	1:100 (FACS)
CD45.2	104	Biolegend	1:100 (FACS)
CXCR5	2G8	BD Biosciences	1:100 (FACS)
Ki67	SolA15	ThermoFisher Scientific	1:100 (FACS)
p-CD3ξ (Y142)	K25-407.69	BD Biosciences	1:400 (1.25 µg/mL) (FACS)
p-ERK1 (T202/Y204)	Polyclonal (#9101)	Cell Signaling Technology	1:50 (FACS)

p-ERK2 (T185/Y187)	Polyclonal (#9101)	Cell Signaling Technology	1:50 (FACS)
p-MPM2 (S/TP motif)	MPM-2	Merck Millipore	1:100 (FACS)
p-ZAP-70 (Y319)	65E4	Cell Signaling Technology	1:400 (FACS)
p-SLC12A2 (T203/T207/T212)	Polyclonal	MRC PPU	1:200 (FACS)
PD-1	29F.1A12	Biolegend	1:100 (FACS)
Mouse IgG (H&L chain)	Donkey polyclonal (highly cross-adsorbed) – AF488	ThermoFisher Scientific	1:500 (FACS)
Rabbit IgG (H&L chain)	Goat polyclonal (highly cross-adsorbed) – AF647	ThermoFisher Scientific	1:500 (FACS)
Sheep IgG	Donkey Polyclonal (highly cross-adsorbed) – CF647	Biotium	1:500 (FACS)
Streptavidin	APC	ThermoFisher Scientific	1:500 (FACS)

Supplementary Data 1. RNAseq data for WNK1-deficient and control CD4$^+$ T cells.
