# Supplementary material for: WNK1-dependent water influx is required for CD4+ T cell activation and T cell-dependent antibody responses
Source: Nat Commun. 2025 Feb 21;16:1857. doi: 10.1038/s41467-025-56778-x (PMC11845700; doi:10.1038/s41467-025-56778-x)
Supplement: Supplementary file 2 — Description of Additional Supplementary Files [file 41467_2025_56778_MOESM2_ESM.pdf]

Description of Additional Supplementary Files

Supplementary Data 1. RNAseq data for WNK1-deficient and control $CD4^+$ T cells.
Excel file containing expression of genes (in transcripts per million, tpm) determined using RNAseq in 12 samples of $CD4^+$ T cells: 6 *Wnk1*$^{+/-}$RCE and 6 *Wnk1*$^{-/-}$RCE.
