# Supplementary material for: WNK1-dependent water influx is required for CD4+ T cell activation and T cell-dependent antibody responses
Source: Nat Commun. 2025 Feb 21;16:1857. doi: 10.1038/s41467-025-56778-x (PMC11845700; doi:10.1038/s41467-025-56778-x)
Supplement: Supplementary file 4 — Reporting Summary [file 41467_2025_56778_MOESM4_ESM.pdf]

nature portfolio

Corresponding author(s): VLJ Tybulewicz

Last updated by author(s): 6/9/2024

Reporting Summary

Nature Portfolio wishes to improve the reproducibility of the work that we publish. This form provides structure for consistency and transparency in reporting. For further information on Nature Portfolio policies, see our Editorial Policies and the Editorial Policy Checklist.

Statistics

For all statistical analyses, confirm that the following items are present in the figure legend, table legend, main text, or Methods section.

n/a	Confirmed	
☐	☒	The exact sample size (*n*) for each experimental group/condition, given as a discrete number and unit of measurement
☐	☒	A statement on whether measurements were taken from distinct samples or whether the same sample was measured repeatedly
☐	☒	The statistical test(s) used AND whether they are one- or two-sided<br>*Only common tests should be described solely by name; describe more complex techniques in the Methods section.*
☒	☐	A description of all covariates tested
☒	☐	A description of any assumptions or corrections, such as tests of normality and adjustment for multiple comparisons
☐	☒	A full description of the statistical parameters including central tendency (e.g. means) or other basic estimates (e.g. regression coefficient) AND variation (e.g. standard deviation) or associated estimates of uncertainty (e.g. confidence intervals)
☐	☒	For null hypothesis testing, the test statistic (e.g. *F*, *t*, *r*) with confidence intervals, effect sizes, degrees of freedom and *P* value noted<br>*Give P values as exact values whenever suitable.*
☒	☐	For Bayesian analysis, information on the choice of priors and Markov chain Monte Carlo settings
☒	☐	For hierarchical and complex designs, identification of the appropriate level for tests and full reporting of outcomes
☒	☐	Estimates of effect sizes (e.g. Cohen's *d*, Pearson's *r*), indicating how they were calculated

Our web collection on statistics for biologists contains articles on many of the points above.

Software and code

Policy information about availability of computer code

Data collection	Flow cytometry data was collected using BD FACSDiva. Western blot data was collected using Image Studio.
Data analysis	Flow cytometry was analyzed using FlowJo (BD) versions 9 and 10. Statistical analysis and data visualization were performed using GraphPad Prism version 8 and Adobe Illustrator.

For manuscripts utilizing custom algorithms or software that are central to the research but not yet described in published literature, software must be made available to editors and reviewers. We strongly encourage code deposition in a community repository (e.g. GitHub). See the Nature Portfolio guidelines for submitting code & software for further information.

Data

Policy information about availability of data

All manuscripts must include a data availability statement. This statement should provide the following information, where applicable:

- Accession codes, unique identifiers, or web links for publicly available datasets
- A description of any restrictions on data availability
- For clinical datasets or third party data, please ensure that the statement adheres to our policy

The data that support the findings of this study are provided in a Source Data file.

Human research participants

Policy information about studies involving human research participants and Sex and Gender in Research.

Reporting on sex and gender	*Use the terms sex (biological attribute) and gender (shaped by social and cultural circumstances) carefully in order to avoid confusing both terms. Indicate if findings apply to only one sex or gender; describe whether sex and gender were considered in study design whether sex and/or gender was determined based on self-reporting or assigned and methods used. Provide in the source data disaggregated sex and gender data where this information has been collected, and consent has been obtained for sharing of individual-level data; provide overall numbers in this Reporting Summary. Please state if this information has not been collected. Report sex- and gender-based analyses where performed, justify reasons for lack of sex- and gender-based analysis.*
Population characteristics	*Describe the covariate-relevant population characteristics of the human research participants (e.g. age, genotypic information, past and current diagnosis and treatment categories). If you filled out the behavioural & social sciences study design questions and have nothing to add here, write "See above."*
Recruitment	*Describe how participants were recruited. Outline any potential self-selection bias or other biases that may be present and how these are likely to impact results.*
Ethics oversight	*Identify the organization(s) that approved the study protocol.*

Note that full information on the approval of the study protocol must also be provided in the manuscript.

Field-specific reporting

Please select the one below that is the best fit for your research. If you are not sure, read the appropriate sections before making your selection.

☒ Life sciences ☐ Behavioural & social sciences ☐ Ecological, evolutionary & environmental sciences

For a reference copy of the document with all sections, see nature.com/documents/nr-reporting-summary-flat.pdf

Life sciences study design

All studies must disclose on these points even when the disclosure is negative.

Sample size	No statistical method was used to predetermine sample size. In vitro and in vivo experiments provide multiple cells from multiple mice for robust analysis.
Data exclusions	For cell cycle and proliferation experiments, dead cells (as determined by LIVE/DEAD Near-IR cell viability dye uptake) were removed.
Replication	All experiments were performed multiple times, and all replication attempts were successful.
Randomization	Experimental animals were not randomized.
Blinding	No blinding was performed.

Reporting for specific materials, systems and methods

We require information from authors about some types of materials, experimental systems and methods used in many studies. Here, indicate whether each material, system or method listed is relevant to your study. If you are not sure if a list item applies to your research, read the appropriate section before selecting a response.

Materials & experimental systems

n/a	Involved in the study	
☐	☒	Antibodies
☒	☐	Eukaryotic cell lines
☒	☐	Palaeontology and archaeology
☐	☒	Animals and other organisms
☒	☐	Clinical data
☒	☐	Dual use research of concern

Methods

n/a	Involved in the study	
☒	☐	ChIP-seq
☐	☒	Flow cytometry
☒	☐	MRI-based neuroimaging

Antibodies

Antibodies used	See table S1.
Validation	ERK 3A7: p44/42 MAPK (Erk1/2) (3A7) Mouse mAb detects endogenous levels of total p44/42 MAP kinase (Erk1/2). It does not cross-react with either JNK/SAPK or p38 MAP kinase. It has been cited in 586 publications.<br><br>OXSR1: polyclonal antibody, has been verified using Western blotting for the correct size. It has been cited in 1 publication.<br><br>p-CHK1 (133D3): Phospho-Chk1 (Ser345) (133D3) Rabbit mAb detects endogenous levels of Chk1 only when phosphorylated at serine 345, validated by Western blotting. It has been cited in 708 publications.<br><br>p-OXSR1: polyclonal antibody, has been verified using Western blotting for correct size, and band intensity is dependent on WNK1 activity. It has been cited in 3 publications.<br>pRPA32 (A300-245A): polyclonal antibody. It has been validated by Fortis Life Sciences according to their standard procedures. It has been cited in 106 publications.<br><br>a-Tubulin (TAT-1): produced in-house at the Francis Crick Institute, derived from commercially available hybridoma cell lines.<br>gH2AX (S139): Recognizes Histone H2A.X phosphorylated at Ser139. It has been cited in 2466 publications.<br><br>CD4 (GK1.5) This antibody was verified by Relative expression to ensure that the antibody binds to the antigen stated. It has been cited in 30 publications.<br><br>CD45.1 (A20): This antibody was verified by Cell treatment to ensure that the antibody binds to the antigen stated. It has been cited in 38 publications.<br><br>CD45.2 (104): This antibody has been reported for use in flow cytometric analysis, immunoprecipitation, and immunohistochemical staining of frozen tissue sections. The 104 antibody does not react with mouse cells expressing the CD45.1 alloantigen. It has been cited in 117 publications.<br><br>CXCR5 (2G8): This antibody has been shown to be reactive to mouse epitopes using QC testing. It has been cited in 7 publications.<br><br>Ki67 (SolA15): This antibody was verified by Cell treatment to ensure that the antibody binds to the antigen stated. It has been cited in 266 publications.<br><br>p-CD3x (Y142) (K25-407.69): This antibody has been cited in 14 publications.<br><br>p-ERK1/p-ERK2 (9101): Phospho-p44/42 MAPK (Erk1/2) (Thr202/Tyr204) Antibody detects endogenous levels of p44 and p42 MAP Kinase (Erk1 and Erk2) when phosphorylated either individually or dually at Thr202 and Tyr204 of Erk1 (Thr185 and Tyr187 of Erk2). The antibody does not cross-react with the corresponding phosphorylated residues of either JNK/SAPK or p38 MAP Kinase, and does not cross-react with non-phosphorylated Erk1/2. This antibody has been cited in 7108 publications.<br><br>p-MPM2 (MPM-2): Anti-phospho-Ser/Thr-Pro MPM-2 Antibody is a Mouse Monoclonal Antibody for detection of phospho-Ser/Thr-Pro and has been tested in ELISA, IHC, IP & WB. This antibody has been cited in 195 publications.<br><br>p-ZAP-70 (Y319) (65E4): Phospho-Zap-70 (Tyr319)/Syk (Tyr352) (65E4) Rabbit mAb detects endogenous levels of Zap-70 only when phosphorylated at Tyr319. This antibody has been cited in 66 publications.<br><br>PD-1 (29F.1A12): This antibody has been verified to react to mouse PD-1, and demonstrated functionally in vivo by blocking of PD-1/PD-L signaling, and in vitro by PD-1 neutralization. It has been cited in 31 publications.

Animals and other research organisms

Policy information about studies involving animals; ARRIVE guidelines recommended for reporting animal research, and Sex and Gender in Research

Laboratory animals	Mice
Wild animals	None
Reporting on sex	Mice were sex-matched within an experiment. Both male and female mice were used; sex-based analysis was not performed.
Field-collected samples	Not applicable
Ethics oversight	All experiments were approved by the Francis Crick Institute Animal Welfare Ethical Review Board and were carried out under the authority of a Project Licence granted by the UK Home Office.

Note that full information on the approval of the study protocol must also be provided in the manuscript.

Flow Cytometry

Plots

Confirm that:

- [x] The axis labels state the marker and fluorochrome used (e.g. CD4-FITC).
- [x] The axis scales are clearly visible. Include numbers along axes only for bottom left plot of group (a 'group' is an analysis of identical markers).
- [x] All plots are contour plots with outliers or pseudocolor plots.
- [x] A numerical value for number of cells or percentage (with statistics) is provided.

Methodology

Sample preparation	Mouse T cells were isolated as described in the Methods section.
Instrument	LSR Fortessa (BD)
Software	FACSDiva for acquisition, FLowJo for analysis and data visualisation
Cell population abundance	No cells were sorted. Routine cell purity exceeded 90% CD4+ T cells following bead-based negative selection.
Gating strategy	All experiments were performed on purified naive CD4+ T cells. Lymphocytes were gated using FSC/SSC, and live cells were selected using LIVE/DEAD near-IR dye. CD4+ T cells were next gated on CD4 levels and further analyzed.

- [x] Tick this box to confirm that a figure exemplifying the gating strategy is provided in the Supplementary Information.
